# Developing a standard dataset in the European registries for rare endocrine and bone conditions—a Melorheostosis dataset

**DOI:** 10.1186/s13023-025-03862-6

**Published:** 2025-07-01

**Authors:** Natasha M. Appelman-Dijkstra, Mariya Cherenko, Gavin P. R. Clunie, Thomas Funck-Brentano, Corinna Grasemann, Adalbert Raimann, Willem F. Lems, Martine Cohen-Solal

**Affiliations:** 1 Department of Medicine, Division of Endocrinology, Leiden University Medical Centre, Albinusdreef 2, Postbox 9600, 2300 RC Leiden, The Netherlands; 2 Metabolic Bone Physician, Cambridge University Hospitals NHS Foundation Trust, Cambridge, UK; 3 Inserm U1132 Bioscar, Université Paris Cité, Centre Viggo Petersen Hôpital Lariboisière, APHP.Nord, 75010 Paris, France; 4 Department of Pediatrics, Ruhr-University Bochum, St. Josef Hospital, Bochum, Germany; 5 Department of Pediatrics & Adolescent Medicine, Division of Pediatric Pulmonology, Allergology and Endocrinology, Medical University Vienna, Vienna, Austria; 6 Department of Rheumatology, Amsterdam University Medical Center, Amsterdam, The Netherlands

**Keywords:** Rare diseases, Registry, Melorheostosis, Minimal dataset

## Abstract

**Background:**

Melorheostosis is a rare skeletal and connective tissue disorder with the estimated prevalence of 1/1,100,000. Low prevalence of rare diseases (RDs) can lead to suboptimal knowledge and expertise among clinicians.

**Methods:**

The European Registries for Rare Endocrine and Bone Conditions (EuRREB) facilitates collection of a set of Core Data Elements and a specific dataset within the ‘condition specific module’ of the Core Registry platform. The Rare Bone Disease Action Group of the European Calcified Tissue Society (ECTS) collaborated with ERN BOND to develop a specific dataset for Melorheostosis.

**Results:**

An initial dataset was shortened to 44 unique variables. In January 2023, the Melorheostosis condition specific module was published and now consists of 18 patients from 2 countries. The median age of patients was 49 years old (range 23–82) and female to male ratio was 15:3 (83.3%). Family history of Melorheostosis was negative for all patients. The most affected bones were lower limbs in 12 cases (66.7%). Specifically, spine, feet and ribs were involved each in 2 cases (11%), skull and pelvis—in one patient each (5.5%). Two patients (11%) suffered from more than 1 lesion. Hyperostosis was present in 3 patients (16.7%), skeletal deformity—in 6 (33%), joint stiffness – in 11 (61%), asymmetry–in 16 (88.9%), joint limitation–in 12 (66.7%) patients. Swelling and muscle atrophy were reported in 1 case each (5.5%), vascular abnormalities—in 2 cases (11%), skin abnormality in 1 case (5.5%). Pain was present in 14 from 18 patients (77.8%). Genetic testing was performed in 5 patients (27.7%).

**Conclusion:**

A condition specific module, for Melorheostosis, within an established registry has been developed. This will serve a useful resource to inform clinicians about this rare disease, and can support several healthcare initiatives such as guidelines creation and healthcare improvement strategies.

## Background

The definition ‘rare disease’ (RD) applies to a multitude of conditions, characterized by great diversity and geographic dispersion. In the EU, the definition of a RD was established in The EU Regulation on orphan medicinal products (1999) as a condition whose prevalence is less than 5 per 10 000 of the population [1]. Accordingly, a conservative, estimate for the population prevalence of RDs is 3.5–5.9%, which equates to 263–446 million persons affected globally at any point in time [2]. However low prevalence of RDs can lead to suboptimal knowledge and expertise among clinicians. One way of improving knowledge in the area of RDs and expertise is the creation of patient registries [3]. For this purpose, in 2016 twenty-four European Reference Networks (ERNs) were created: among them—the European Reference Network on rare endocrine conditions (Endo-ERN) (https://endo-ern.eu/) and the European Reference Network for Rare Bone Diseases (ERN BOND) (https://ernbond.eu/) [4, 5]. One of the most important aims of ERNs is to facilitate improvements in the quality of health care across the EU and elsewhere. For this purpose, the EU supported the creation of registries to support the ERNs in their aim. Registries can provide data derived from ‘real world’ health care settings to inform analyses of RD epidemiology, natural history, clinical practice, patient outcomes, quality of life and treatment efficacy and safety. In 2020, The European Registries for rare bone and mineral conditions (EuRR-Bone), the RD registry linked to ERN BOND was funded and implemented. EuRR-Bone was linked to both ERN BOND and Endo-ERN in 2020 with platform project teams merged under the name EuRREB, thus serving the needs of the two ERNs and reflecting a broader stakeholder community including scientific societies like the European Calcified Tissue Society (ECTS). Concurrently ECTS and ERN BOND liaised through the work of ECTS’s Rare Bone Disease Action Group (RBDAG) with an aim of promoting work on developing core RD datasets. One of these conditions was Melorheostosis (ORPHAcode 2485).

Melorheostosis is a rare skeletal and connective tissue disorder characterized by a sclerosing bone dysplasia, usually limited to one side of the body, sometimes monostotic, that can manifest with pain, stiffness, joint contractures and deformities The estimated prevalence is 1/1,100,000 with > 400 cases reported in literature so far [6]. Melorheostosis is probably not a hereditary disease but is associated with random somatic mutations related to MAP2K1 [7–9]. The diagnosis is usually made radiologically, by the classical “dripping candle wax” sign although biopsy can be done to rule out other conditions, especially where there is concern over skeletal malignancy [9–12]. The long bones of the lower extremities are most typically affected, but any bone can be involved [11, 13]. The condition maybe disclosed serendipitously in late adolescence or early adulthood but can also present with pain. An association with skin changes and vascular involvement has been reported [11, 12, 14, 15]. The main clinical morbidity of Melorheostosis is pain and to date there is no specific treatment available nor are there any guidelines or standards of care addressing the management of patients with Melorheostosis [9, 11, 12]. Additionally, long-term data on the natural course of the condition are lacking. The main purpose of this study was to create a dataset and the registry on Melorheostosis to fill the gap in the knowledge on this rare condition. The multicentre cohort of patients in this registry can be a perfect background in the future for guidelines creation and healthcare improvement strategies.

## Methods

### e-REC

EuRREB consists of 2 registries, an electronic REporting of Conditions platform (e-REC), and the Core registry. e-REC collects information about the monthly number of new cases seen in reporting centres, facilitating the mapping of expert centres and supporting the continuous monitoring program of the ERNs since the mapping is performed using ORPHA codes. The full data dictionary of e-REC is available online (https://eurreb.eu/registries/data-dictionaries/). A comprehensive description of the methods of the registries has been previously published [16].

By October 2024, a total of 108 centres from 31 countries reported a total of 45,476 new cases using e-REC (https://eurreca.lumc.nl/ERec/Account/Login). Of these 108 centres, fifty-seven are members of Endo-ERN only, 28 of Endo-ERN and ERN-BOND, three of ERN-BOND only and 20 expert centres are not affiliated to any ERN. A total of 1932 new cases of rare bone disorders have been reported in e-REC, among them 50 cases of Primary bone dysplasia with increased bone density, the ORPHA code under which Melorheostosis is listed.

### The core registry

The registries second platform is the Core registry which allows recording of more case details and thus requires patient informed consent and local ethical approvals for participating centres. The Core registry collects 15 elements of the Common Data Element set for rare disease registration as recommended by the Joint Research Centre (https://eu-rd-platform.jrc.ec.europa.eu/set-of-common-data-elements_en). The Core registry allows collection of more specific diagnoses and condition specific modules. The data dictionary and its modules are openly available at https://eurreb.eu/registries/data-dictionaries/.

**Fig. 1:**
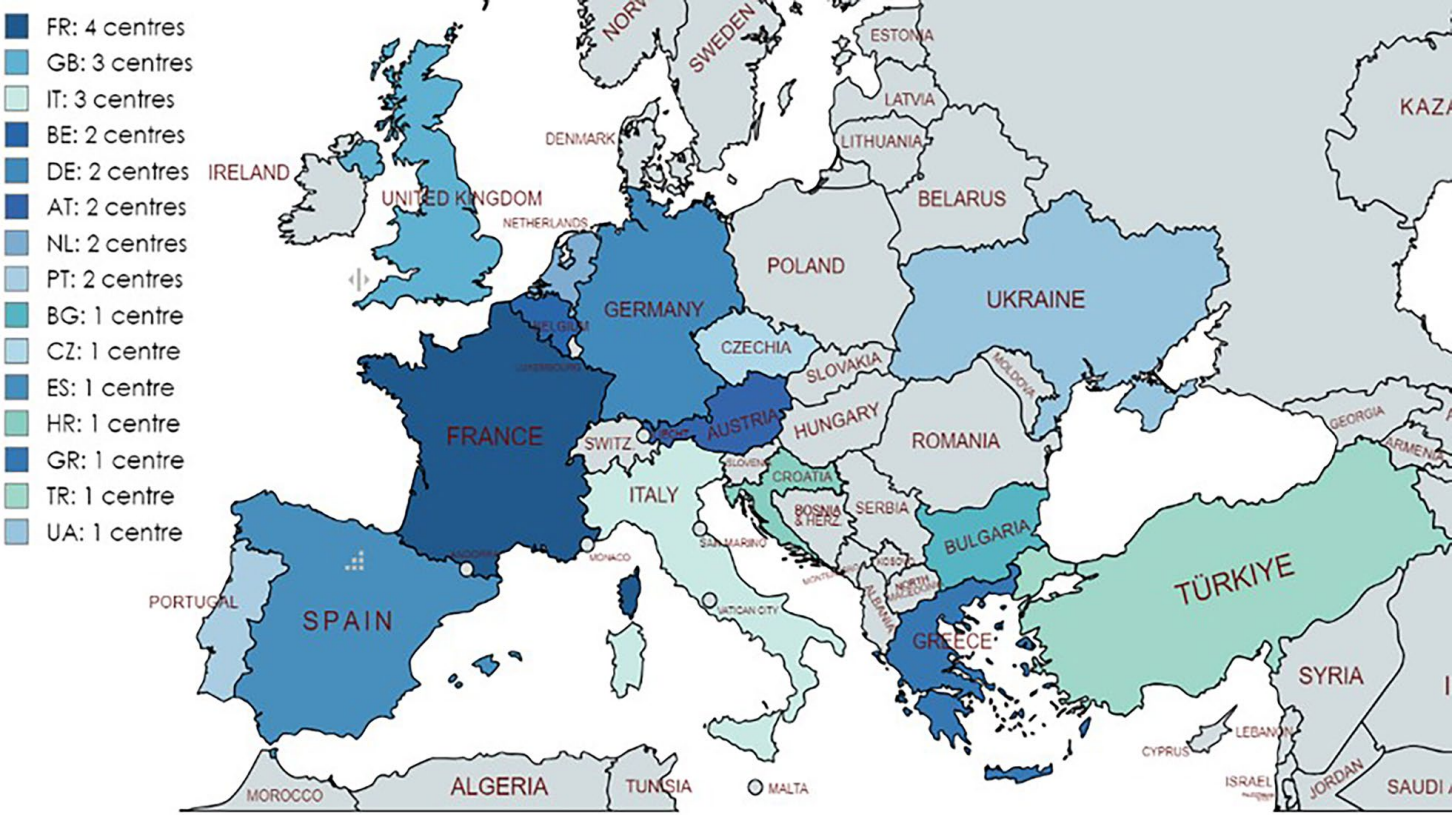
Reporting countries and centres in the Core Registry for Bone and Mineral Conditions

By November 2024, 27 centres from fifteen different countries registered 1348 patients with rare bone and mineral conditions in the Core Registry. Of these twenty-seven centres, 17 are affiliated to Endo-ERN, ERN BOND or both, and 10 are not affiliated to any ERN, see Fig. 1.

### PROMs

In addition, the Core Registry platform allows patients to have access to their own data and to contribute Patient Reported Outcome Measures (PROMs) data. The Core registry allows collection of EQ5D data, but for bone RDs it was decided to add 3 specific PROMs: the Brief Pain Inventory Short Form (BPI-SF), the World Health Organisation Classification on Mobility (WHO-ICF mobility) and the Musculoskeletal Health Questionnaire (MSK-HQ). The type of PROMS was decided based on availability eg validated in many languages, validated and either free of charge or with a single low price for the license as well as complaints (pain, quality of life). BPI-SF recently has become one of the most widely used measurement tools for assessing clinical pain. First used for oncology patients, BPI-SF was subsequently validated for non-oncological pain [17–19] and is available in 11 languages on the EuRREB platform. The WHO-ICF mobility questionnaire consists of 14 questions scoring different aspects of mobility, such as daily routine, sitting, running, walking, driving etc., each with 7 levels of impairment scored (from ‘no difficulty’ to ‘complete difficulty’) and has been validated in different skeletal dysplasias [20, 21]. MSK-HQ is a short questionnaire that allows people with musculoskeletal conditions (such as arthritis or back pain) to report their symptoms and quality of life in a standardized way. This questionnaire is often used to evaluate the effects of treatment (monitoring interindividual patients’ results before and after treatment initiation or titration). MSK-HQ has been validated for different muskuloskeletal disorders and is available in 5 languages on the EuRREB platform [22].

### Condition-specific modules

Within the Core Registry, condition-specific modules (CSMs) can be created. The condition specific modules are created by a group of experts in a specialist field and are specific datasets for a particular condition. Currently the Core Registry supports 11 CSMs, six of which pertain to rare bone diseases. The Rare Bone Disease Action Group (RBDAG) of the European Calcified Tissue Society (ECTS) has collaborated with ERN BOND to develop a condition-specific module on Melorheostosis.

#### Melorheostosis condition-specific module development

The main objective of the Melorheostosis condition-specific module was to define a minimum dataset (MDS) that could be derived during routine clinical practice throughout EU and beyond. We reasoned that confining data contributions within a minimum dataset would minimise the workload for participating centres and improve quality of data collection.

A condition-specific module study group was formed by members of the ECTS RBDAG, the coordinator of ERN BOND and the EuRREB project team. The group consisted of 10 experts (6 endocrinologists and 4 rheumatologists) in the field of bone dysplasia from 10 different expert centres from 6 countries (The United Kingdom, the Netherlands, France, Germany, Denmark and Italy). To derive a minimum dataset the following process was undertaken: a comprehensive list of data fields was generated in preparation by clinicians (study group members) during routine clinical monitoring for patients with Melorheostosis. Also, a list of data fields from known local or national surveillance registries were compiled. Each participant of the condition-specific module study group was asked to determine the importance of each data field (the importance of each data field referring to the considered relative importance of the information to enable good clinical care of patient and in establishing the diagnosis and assessing the natural course of disease). The ease of data collection was based on the experts’ experience in how readily available the information was in routine clinical settings. When developed the full dataset was discussed within the study group and only variables that were agreed on by all were included. The first variable list was drafted by one of the expert centers as it was used for their local database. Also, the condition-specific module study group has reached consensus on the patient reported outcome measures (PROMS) in pain and mobility assessment. Preparations for the development of the finalised minimum dataset took place over a total of 12 months, a time in which there were 4 teleconference meetings between condition-specific module study group members (where the results of the 2 rounds of voting were analysed).

## Results

Consensus discussions derived a primary dataset, which was then reduced to 44 unique variables. Ten variables (date of birth, sex, country of birth and residence, date of first clinical manifestation and date of diagnosis, participation in detailed disease registry, availability of biobank samples, follow-up status and date of death) were already collected as part of the common data elements set and are already part of the registry. The condition specific module Study Group agreed to divide case data recording under the headings: general, clinical features, genetic analysis, fractures, surgery and medication.

The specific Dataset agreed for the Melorheostosis CSM is shown in Table 1. The full condition specific module data dictionary is available online at https://eurreb.eu/registries/data-dictionaries/

**Table 1:** The Dataset for Melorheostosis

General	Family ID, Proband**,** Family history
Clinical features	Height (cm) Weight (kg), X-ray features, Hyperostosis, Involved site Skeletal dysplasia (deformity), Joint stiffness Asymmetry, Joint limitation of affected site Swelling, Muscle atrophy, Abnormality of the skin, Skin abnormalities details, Vascular abnormalities, Pain, Site involved by pain, Pain adult (VAS), Pain—child (Wong-Baker FACES scale)
Genetic analysis	Genetic diagnosis germline, DNA c, Protein c, Mutation type, Genetic diagnosis somatic
Fractures	Has the patient experienced fractures (over the last year)? If yes, was it the fracture at affected site? Involved site
Surgery*	Date of intervention, Location, Indication, Type of intervention
Medication*	Start date, Stop date, Bone active medication, Pain medication, Has the patient developed medication related adverse events? Please, indicate the medication, Adverse event, Was it the reason to stop the medication?

^*^Same questions are repeated several times (for several interventions, medications, adverse events)

**Fig. 2:**
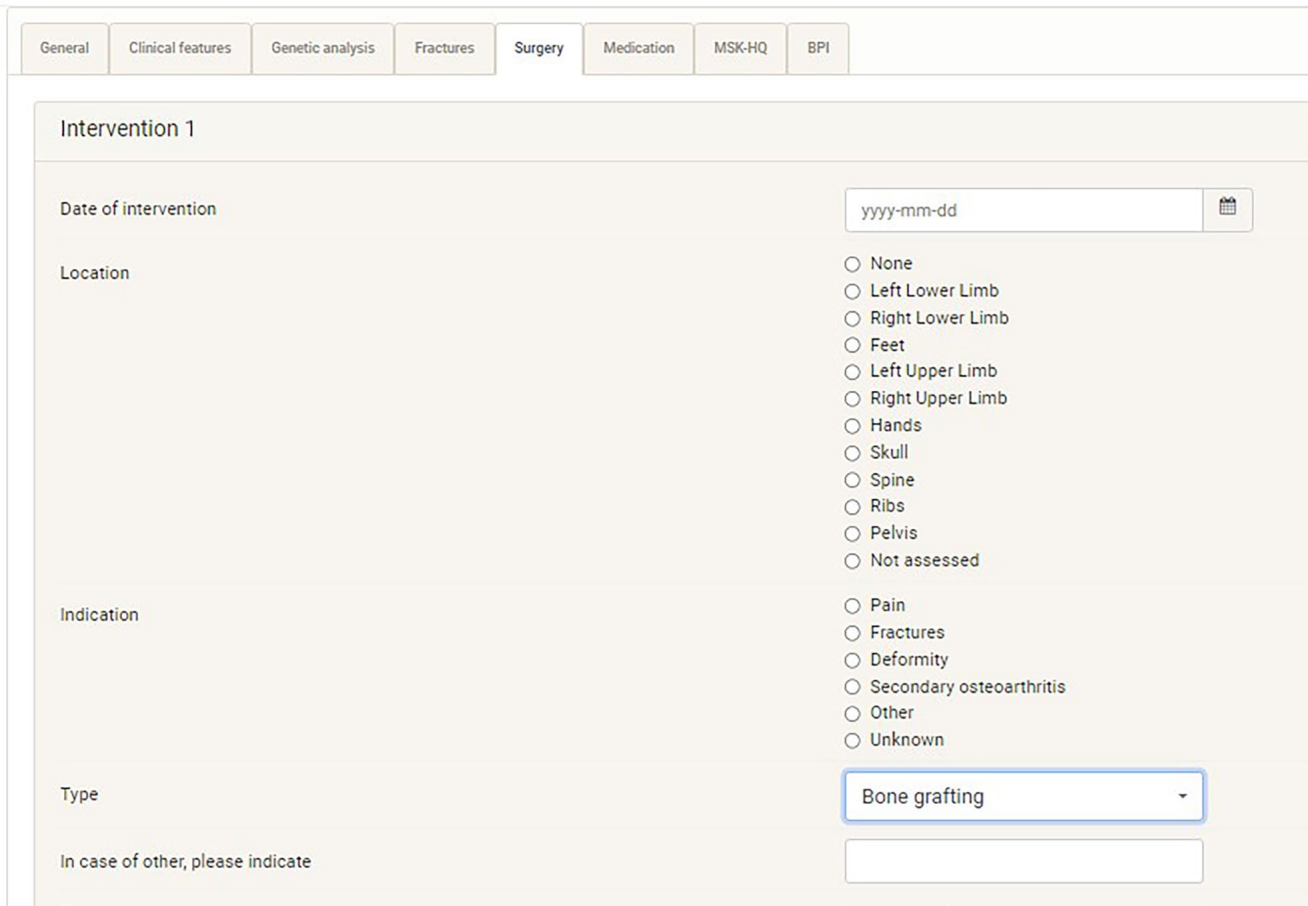
Melorheostosis module appearance on the Core registry platform

To simplify data collection most of the answers required in the CSM are of predefined case features. But free text answers can also be given to enhance further data collection (Fig. 2).

The first draft of the condition specific module has been created as an Excel file and has been revised according to ongoing condition specific module study group discussion in due course (for 2 months). Then a beta version of the condition specific module has been built on the Core registry platform for one month and has been tested for 3 months by the condition specific module group. Multiple timepoints for data entry are intended with different dates of assessment possible, allowing both recording of case history data and data prospectively collected within a condition Specific Module. All the data entries could be amended and deleted by the clinician, which will be logged automatically by the system.

As one of the main clinical features of Melorheostosis is pain, the condition specific module group agreed that pain monitoring would be by a Visual Analogue Scale (VAS) score for adults, and The Wong-Baker Faces Pain Rating Scale score—for children over 3 years old [23, 24] and BPI-SF.

In January 2023, the Melorheostosis condition specific module was activated. ERN BOND, ECTS and EuRREB included the module launch repeatedly in their newsletters. The Melorheostosis condition specific module was also advertised during the annual ECTS meetings in 2023 and 2024, in relevant webinars and on ECTS social media. On repeating testing and feedback, on average it takes less than 10 min to fill-in all data entry fields for the whole Melorheostosis condition specific module.

## Condition specific module data entries to date

At time of writing, the Core registry contains 20 cases of Melorheostosis (among them one case combined with Osteopoikilosis) from two countries (expert centres). The median age of cases is 49 years old (range 23–82), with females were more prevalent (85%). The median age of first presentation was 34 years old (range 9–69) and median age at diagnosis was 33.5 years old (range 9–69), and time to diagnosis varied from 0 to 168 months (mean 16 ± 40). Biobank sample available for 6 patients (30%). The diagnosis for all of them was confirmed based on the characteristics of the radiographs by multidisciplinary team including bone experts.

For 18/20 patients (90%) the Melorheostosis CSM was completed. Median age was 49 years (range 23–82); and female to male ratio was 15:3 (83.3%). None of the patients had a family history for Melorheostosis. Clinical and radiological findings of the 18 patients are shown in the Table 2. Sixteen out of eighteen patients (89%) were recorded as having monostotic disease. The most frequently affected sites were the lower limbs 12/18 cases (67%). Spine, feet and ribs were involved each in 2 cases, skull and pelvis—in one patient each. Two patients (11%) suffered from more than 1 lesion. Hyperostosis was present in 3 patients (16.7%), skeletal deformity was reported in 6 patients (33%), joint stiffness–in 11 patients (61%), asymmetry–in 16 patients (88.9%), joint limitation–in 12 patients (66.7%), swelling and muscle atrophy were reported in 1 case each (5.5%). Vascular abnormalities were reported in 2 patients (11%) and a skin abnormality (white nevus) in 1 case (5.5%). Pain was reported in 14/18 patients (78%) but was assessed by Pain VAS and BPI-SF in two patients only (11%). Genetic testing was performed in five patients (28%) but yielded no mutation. No skin biopsy was performed in the patient with skin involvement.

**Table 2:** Clinical and radiological findings of Melorheostosis patients in the condition specific modules (data as of November 2024)

Clinical and radiological findings		Number of patients, n (%)
Females/Males		15 (84%)/3 (16%)
Affected site	Lower limbs: Upper leg Lower leg Feet	12 (67%) 1 (6%) 11 (61%) 2 (11%)
	Spine	2 (11.1%)
	Ribs	2 (11.1%)
	Skull	1 (5.5%)
	Pelvis	1 (5.5%)
Polyostotic disease		2 (11.1%)
Pain		14 (78%)
Hyperostosis		3 (17%),
Skeletal deformity		6 (34%)
Joint stiffness		11 (61%)
Joint mobility limitation		12 (67%)
Asymmetry of bones and joints		16 (89%)
Swelling of joint		1 (5%)
Muscle atrophy		1 (5%)
Vascular abnormalities		1 (5%)
Skin abnormality		1 (5%)

## Discussion

In comparison to other studies on Melorheostosis [10, 12] data recorded on our CSM so far confirms a strong female prevalence of Melorheostosis and first clinical symptoms manifesting in adulthood. Our data validate that pain is one of the most prevalent symptoms (78% in our case series), as well as asymmetry of bones and joints lesions (89%) and joint movement limitation (67%) and that the most commonly affected bones are in lower limbs (67%). Accordingly, we can say already from our, and others’ data, that there are important lessons for healthcare needs involving input from lower limb physical therapists and orthotists.

We present here the development and implementation of the first prospective international registry on Melorheostosis and provide some insights on data recording in the first-year. The derivation of a *minimal* data set, which has been shown in general to decrease time taken for data entry [26], we think, likely facilitated good case data entry in our Melorheostosis CSM.

Normally, the advised main goal of registry construction is to choose the most important parameters to collect, using predefined answers and not to operate with a too large dataset, as we have done here [25, 27]. The most successful registries are known to operate using fewer, rather than more, parameters, and as the consequence less data are missing [25, 27]. In general, deriving registry minimal datasets typically starts with multiple parameter data fields requested by experts, but then the dataset is subsequently refined and reduced, to focus only on the most essential data from clinical practice.

After the dataset has been established and the registry is published the data fields still need to be filled, creating the final challenge any registry enabling human resources to enter data. So far, expert centres have mostly been focussing on clinical pathways in rare diseases while data collection is not a standardised part of rare disease care.

Our Melorheostosis CSM is streamlined to facilitate repeated data entry. In rare conditions like Melorheostosis knowledge on the natural course of disease is lacking and there are no therapies available which influences patients outcomes. Continuous data entry into the Melorheostosis CSM we feel, will allow for data entry on ongoing aspects of care including therapy. In time we think such data will be important and useful for patients, clinicians and RD organisations as a resource with which to plan further research and even therapy trials. In addition, it is clear to us that prospective data entry facility, as we have set up here, will help clinicians and Health Care managers improve Melorheostosis, and other RD, care pathways and can even help inform costs of care (for example recording effective and ineffective therapeutic approaches). The CSM sits within a wider registry infrastructure which enhances the ability of local sites in different countries to get relevant regulatory approvals to contribute. It is important to emphasize that EuRREB is open to all clinical centres, regardless of their ERN affiliation and geographical position. This borderless approach facilitates wider data collection throughout the world, works against data biases (limited case selection based on Demographic and Geographical constraints) and, we think, can improve opportunities to compare disease and disease management differences in different parts of the world. EuRREB caters the needs of experts in different fields, various medical and patients’ societies and is open to collaboration with different stakeholders. An important part of the Core registry is the ability of patients to directly access their data, particularly to collect PROM data. However, as it is crucial to collect PROMs data in specific time period: automatic reminders are sent to patients, to complete their data entry, to decrease the quantity of missing data. It’s noteworthy that in our Melorheostosis condition specific module, PROMs are translated to different languages to facilitate data return across the breadth of EU countries. Registry team actively engages with clinicians and patients inputting data via regular online sessions, specific guides on how to use CSM and annual symposiums (with live training sessions).

## Limitations and future perspectives

In addressing biases of our CSM for Melorheostosis, we acknowledge that patients referred to specialist centres contributing data collection may be the most symptomatic patients and that data from asymptomatic or minimally symptomatic patients may not be captured on the Melorheostosis CSM. Such case ascertainment bias might be relevant in interpreting Melorheostosis gender prevalence. Our hope is that case data entry on the CSM increases. Most of published case series to date, include approximately 20 patients (range 19–24). We believe that expanding our registry and collaborating with others will greatly improve data our knowledge about Melorheostosis [10, 12].

## Conclusion on datasets

Our aim is to collect prospective ongoing data and increase contribution across a wide geographical area, in other words to look at the natural history of the disease. It’s also important to emphasize that the dataset can harbour more studies, so more experts are invited to develop their own research question. Such multicentre international case and data accrual on Melorheostosis might to drive consensus opinions of disease management, on standards of care and guidelines creation and in informing changes in healthcare for people living with Melorheostosis.

## Data Availability

The data supporting this study’s findings are not openly available due to reasons of sensitivity and are available from the corresponding author upon reasonable request.

## Abbreviations

EuRREB: European Registries for Rare Endocrine and Bone Conditions
ERNs: European Reference Networks
ERN BOND: European Reference Network for Rare Bone Diseases
Endo-ERN: European Reference Network on Rare Endocrine Conditions
ECTS: European Calcified Tissue Society
RD: Rare disease
RBDAG: The Rare Bone Disease Action Group
PROMs: Patient Reported Outcome Measures
BPI-SF: The Brief Pain Inventory Short Form
WHO-ICF mobility: The World Health Organisation Classification on Mobility
MSK-HQ: The Musculoskeletal Health Questionnaire
CSMs: Condition-specific modules
MDS: Minimum dataset
